# Independent cancer research in low- and middle-income countries: why it matters and how to build it

**DOI:** 10.1093/oncolo/oyag356

**Published:** 2026-09-07

**Authors:** Oscar Arrieta, Eduardo Rios-Garcia, Cittim B Palomares-Palomares, Alejandro Aranda-Gutierrez, Regina Barragan-Carrillo, Carlos H Barrios, Andrés F Cardona, Luis Corrales, Narjust Florez, Juan-Manuel Hernandez-Martinez, Claudio Martin, Jackson Orem, Luis Eduardo Pino, Ricardo Plancarte, Gustavo Werutsky, C S Pramesh

**Affiliations:** Thoracic Oncology Unit, Instituto Nacional de Cancerología (INCan), Mexico City, 14080, Mexico; Thoracic Oncology Unit, Instituto Nacional de Cancerología (INCan), Mexico City, 14080, Mexico; Centro de Investigación en Ciencias de la Salud (CICSA), Facultad de Ciencias de la Salud, Universidad Anáhuac México Norte, Huixquilucan, Estado de México, 52786, Mexico; Thoracic Oncology Unit, Instituto Nacional de Cancerología (INCan), Mexico City, 14080, Mexico; Facultad de Ciencias Administrativas y Sociales, Universidad Autónoma de Baja California (UABC), Ensenada, Baja California, 22860, Mexico; Departamento de Hemato-Oncología, Instituto Nacional de Ciencias Médicas y Nutrición Salvador Zubirán, Mexico City, 14080, Mexico; Medical Oncology Department, Instituto Nacional de Cancerología (INCan), Mexico City, 14080, Mexico; Latin American Cooperative Oncology Group (LACOG), Centro de Pesquisa em Oncologia, Hospital São Lucas PUCRS, Porto Alegre, 90610, Brazil; Thoracic Oncology Unit and Direction of Research and Education, Luis Carlos Sarmiento Angulo Cancer Treatment and Research Center (CTIC), Bogotá, 110131, Colombia; Department of Medical Oncology, Centro de Investigación y Manejo del Cáncer (CIMCA), San José, 10103, Costa Rica; Lowe Center for Thoracic Oncology, Dana-Farber Cancer Institute, Harvard Medical School, Boston, MA 02215, United States; Thoracic Oncology Unit, Instituto Nacional de Cancerología (INCan), Mexico City, 14080, Mexico; Thoracic Oncology Unit, Alexander Fleming InstituteBuenos Aires, C1426, Argentina; Uganda Cancer Institute, Kampala, 3935, Uganda; Clinical Oncology Department, Carlos Ardila Lülle Cancer Institute-ICCAL, Fundación Santa Fe de Bogotá, Bogotá, 110111, Colombia; Pain Clinic, Instituto Nacional de Cancerología (INCan), Mexico City, 14080, Mexico; Latin American Cooperative Oncology Group (LACOG), Porto Alegre-RS, 90619, Brazil; Tata Memorial Centre, National Cancer Grid (India), Homi Bhabha National Institute, Mumbai, 400012, India

**Keywords:** low- and middle-income countries, cancer research, investigator-initiated trials, health equity, population-based cancer registries, pragmatic clinical trials

## Abstract

By 2030, an estimated 75% of cancer deaths will occur in low- and middle-income countries (LMICs). Weak surveillance systems and limited research infrastructure often hide the true magnitude of this burden. Thus, independent, locally led research is essential for generating high-quality data, addressing regional disease patterns, and informing context-specific strategies for cancer control. Low national investment, heavy reliance on industry-sponsored trials, and a scarcity of trained clinician scientists hinder independent cancer research in LMICs. In this narrative review, we synthesize the published literature and present a descriptive analysis of oncology trials registered on ClinicalTrials.gov (accessed July 2025), categorized by country, funding source, and tumor site and normalized to disease burden, drawing on illustrative examples from Africa, Asia, and Latin America. We highlight the transformative impact of context-sensitive research initiatives, explore disparities in sponsorship by country and tumor type, and outline ways to strengthen the research infrastructure. These include the establishment of population-based cancer registries, regional funding mechanisms, streamlined regulatory frameworks, and workforce development through formal research training and protected time. Finally, we propose strategies to enhance international collaboration and amplify LMIC participation in setting global cancer research priorities.

Implications for Practice In low- and middle-income countries (LMICs), most cancer clinical trials are industry-sponsored, leaving locally relevant questions, such as, treatment de-escalation, dose optimization, cost-effectiveness, and implementation, largely unanswered. Independent, locally led research is essential to generate context-specific, decision-grade evidence and has already reshaped national policy. For clinicians and policymakers, practical levers include establishing population-based cancer registries, securing regional and public research funding, streamlining ethics review, protecting clinician-scientists’ research time, and adopting pragmatic trial designs that test real-world effectiveness at lower cost. Strengthening these foundations would align evidence generation with the actual needs of LMIC health systems and advance more equitable cancer care.

## Cancer today

As global life expectancy has increased, the incidence and mortality of neoplastic diseases have risen in parallel. Low- and middle-income countries (LMICs) are experiencing rising cancer incidence in line with global trends; however, they face a disproportionately high share of cancer mortality. Projections indicate that by 2030, approximately 75% of all cancer deaths will occur in LMICs, highlighting the growing and unequal burden in these regions.^1^^,^^2^

In oncology, research plays a critical role in improving the outcomes and quality of life of patients. It deepens our understanding of disease biology, optimizes existing therapies, and drives the development of novel treatment strategies. Moreover, cancer research contributes to the identification of risk factors, supports early detection efforts, and informs the creation of evidence-based public health policies.^3^

Unfortunately, several barriers hinder independent cancer research in LMICs. These include limited national investment in research and development, underfunded academic institutions, scarcity of trained clinician-scientists, absence of protected research time, and the predominance of industry-sponsored trials (ISTs), whose objectives do not always align with regional health priorities. Collectively, these constraints limit the generation of locally relevant evidence, weaken the foundation for effective cancer control policies, and reduce opportunities for innovation.

As a narrative review, the initiatives presented here are illustrative rather than exhaustive. We organize our discussion around 3 LMIC regions: Latin America (Brazil, Mexico, Colombia, Costa Rica, and Argentina), Asia (India), and sub-Saharan Africa (Uganda and Kenya), chosen to reflect the regional expertise and clinical settings of our international author group, several of whom lead cancer research programs in these countries. From each, we draw on initiatives that have informed local policy or clinical practice.

## National investment and the value of locally led research

Research activity is widely considered a reliable indicator of economic development and societal progress. The United States, through the National Cancer Institute (NCI), has demonstrated how sustained public investment shapes national research ecosystems. In fiscal year 2020, the NCI’s annual budget was approximately US $6.25 billion, rising to roughly US $7.2 billion by fiscal year 2024, reflecting a sustained national commitment to cancer research.^4^

A pivotal example of the output of sustained investment is the 2004 discovery, supported by the US National Institutes of Health, that dramatic responses to gefitinib in non–small-cell lung cancer (NSCLC) were driven by activating mutations in the EGFR gene.^5^ Although earlier milestones, notably imatinib in chronic myeloid leukemia, had already established the promise of molecularly targeted therapy,^6^ this breakthrough was a defining advance in precision oncology for solid tumors and helped usher in an era of personalized medicine. Since then, targeted therapies have transformed the treatment landscape across multiple malignancies, delivering substantial improvements in patient outcomes worldwide.^7^

**Figure 1.**
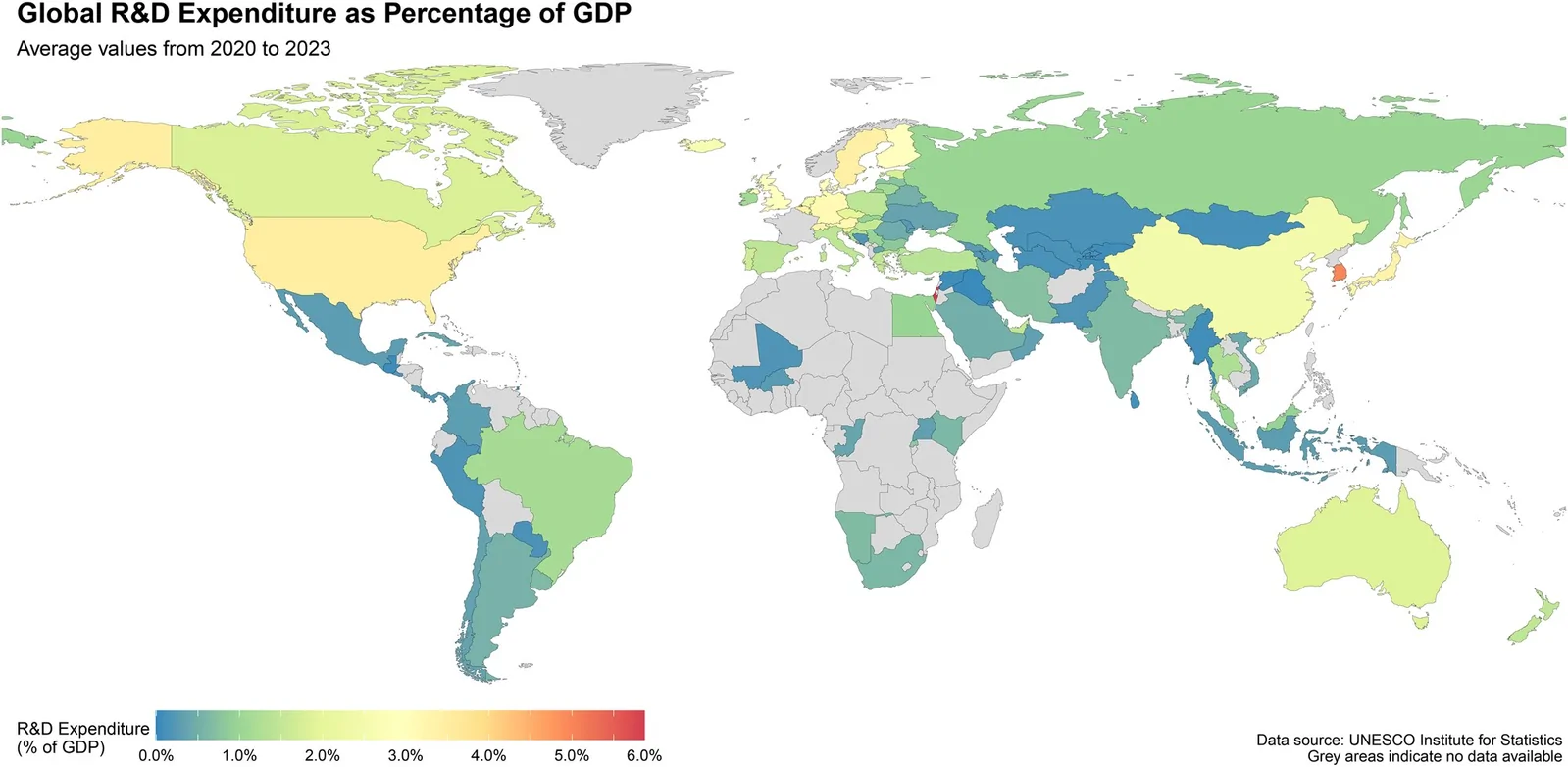
Global gross domestic expenditure on research and development (GERD) as a percentage of GDP, averaged across 2020-2023. Source: UNESCO Institute for Statistics; https://databrowser.uis.unesco.org/, accessed September 11, 2025.

These advances highlight what is possible when research infrastructure aligns with national priorities. However, this contrasts with LMICs, where underinvestment in research remains a barrier. High-income countries invest approximately 3% of GDP in R&D, while most LMICs remain below 1.5%, as seen in Uganda, Brazil, India, and Mexico^8^ (Figure 1). This gap correlates with differences in cancer outcomes and health system performance.^9^ Limited investment prevents LMICs from conducting impactful research or improving patient care, with research often directed by foreign institutions rather than local needs. Venezuela exemplifies how economic crisis can degrade oncology care and research capacity, with shortages of essential cancer medicines and the emigration of specialists eroding services that were once among the region’s strongest.^10^

Despite these challenges, some LMICs have shown that strategic research investment can yield lasting benefits. For comparison, the United States published 34,674 cancer-related articles over 5 years.^11^ Between 2000 and 2018, Brazil produced 3565 articles (41.8% of Latin America’s output), while Mexico published 1412 peer-reviewed cancer articles (16.6%). These counts are based on PubMed-indexed publications and therefore likely underestimate Latin American output, since many Spanish- and Portuguese-language regional journals are not indexed in PubMed. Latin American researchers have nonetheless generated impactful clinical findings. A key study found a 31.2% prevalence of EGFR mutations in Mexican NSCLC patients, higher than in European populations.^12^ This discovery led the Mexican government to fund molecular diagnostic testing and anti-EGFR therapies in the public health system.

This experience underscores the significance of independent, locally driven research in shaping national policy and advancing equity in cancer care. It also highlights the importance of tailoring research plans to reflect local epidemiology, rather than relying solely on imported data from high-income settings. Notably, the success of this initiative demonstrates that strategic, problem-focused research can yield direct patient benefits and inform sustainable health system reforms.

## Challenges in conducting investigator-initiated trials in LMICs

Clinical trials are essential for generating new evidence across multiple domains of oncology, including drug development, diagnostics, and molecular epidemiology. However, these trials require a significant investment of human capital and infrastructure. In many LMICs, the absence of dedicated governmental support has made it difficult to establish investigator-initiated trial (IIT) programs. Thus, substantial funding and infrastructure have been catalyzed by pharmaceutical partners,^13^ yet some locally prioritized questions, such as de-escalation, implementation, or cost-effectiveness, are better addressed by publicly funded IITs.

The value of publicly funded IITs is evident when findings yield clinical or economic benefits. In high-income settings the SONIA trial showed that, in hormone receptor–positive, HER2-negative advanced breast cancer, deferring CDK4/6 inhibitors to second-line therapy maintained progression-free survival while substantially lowering treatment costs, driven by shorter CDK4/6-inhibitor exposure.^14^^,^^15^ In LMICs, the Mexican ALEK-B study combined bevacizumab with alectinib in ALK-rearranged NSCLC, achieving 97% and 64% progression-free survival at 12 and 36 months with a 100% response rate.^16^ Together, these IITs, one from a high-income setting, one from an LMIC, show how independent research can refine treatment efficacy and, where it directly addresses cost as in SONIA, improve affordability, complementing industry-sponsored research.

To complement the narrative synthesis, we analyzed oncology trials registered on ClinicalTrials.gov (accessed July 10, 2025). Records were tabulated by country, tumor site, and funding source. Trials were classified as industry-sponsored (ISTs) when the registry “Funder Type” was Industry, and as investigator-initiated (IITs) when the funder was governmental, academic, or another non-industry sponsor. Five common solid tumors (breast, lung, prostate, colorectal, and melanoma) were selected a priori as comparators. The analysis is descriptive only; counts and proportions are reported without inferential statistical testing. To enable equitable cross-country comparison, trial counts were additionally normalized to national cancer burden using GLOBOCAN 2022 estimates and expressed as trials per 1000 incident cases and per 1000 cancer deaths.^2^ Because ClinicalTrials.gov predominantly captures interventional studies and may not include trials registered solely in regional or WHO ICTRP–linked registries, registry-based and population-based research are underrepresented.

**Figure 2.**
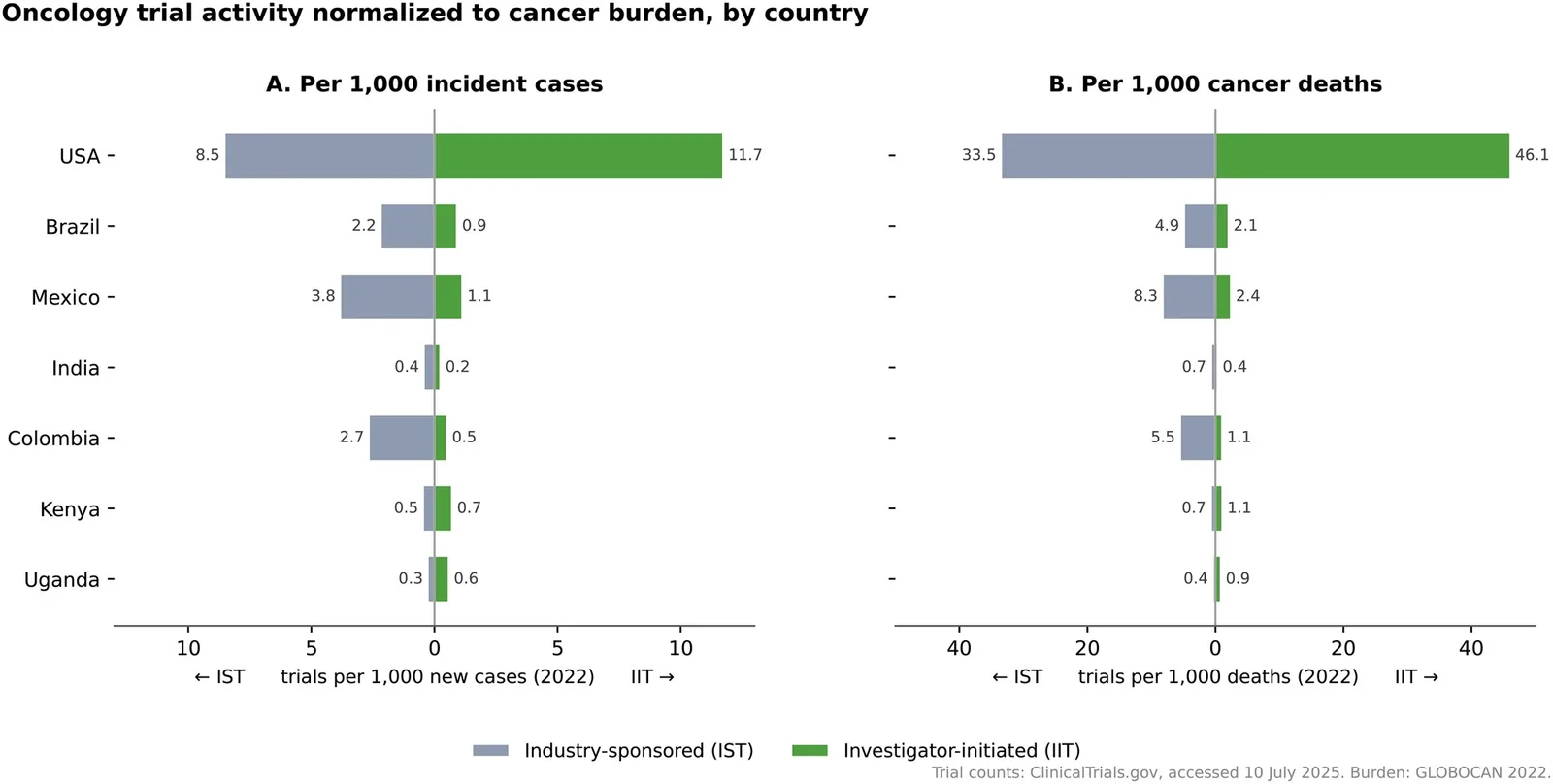
Registered oncology trials normalized to cancer burden, by country: trials per 1000 (A) incident cases and (B) cancer deaths, with industry-sponsored (IST) left and investigator-initiated (IIT) right of a zero axis. Counts are cumulative ClinicalTrials.gov registrations (accessed July 10, 2025); denominators are GLOBOCAN 2022 all-cancer estimates.^2^ Values index relative research intensity, not per-patient probability.

Despite the benefits of IITs and academic research, industry funding dominates clinical trials, leaving context-specific questions unanswered. Of 115,762 cancer trials in the registry, 48,223 are US-based, of which 42.1% are industry-funded. Patterns differ in LMICs: in Latin America, ISTs account for 70.5% of cancer trials in Brazil (1369/1943) and 77.2% in Mexico (794/1029). In India, 64.7% of 959 oncology trials were industry-sponsored. In sub-Saharan Africa, Kenya and Uganda host far fewer cancer trials overall (53 and 31, respectively), with industry sponsoring under 40%. This lower share more likely reflects limited commercial interest in these markets than a deliberate shift toward publicly funded research, highlighting an overall scarcity of cancer trials even as it points to an opportunity to build public research capacity where industry interest is low. Even after adjusting for disease burden, the disparity persisted: the United States registered roughly 20 oncology trials per 1000 incident cases (about 80 per 1000 cancer deaths), versus no more than about 5 per 1000 incident cases in every Latin American, Asian, and sub-Saharan African country examined. The gap was widest for investigator-initiated trials, ranging from about 0.2 to 1.1 per 1000 incident cases across these LMICs vs 11.7 in the United States **(**Figure 2**)**.

**Figure 3.**
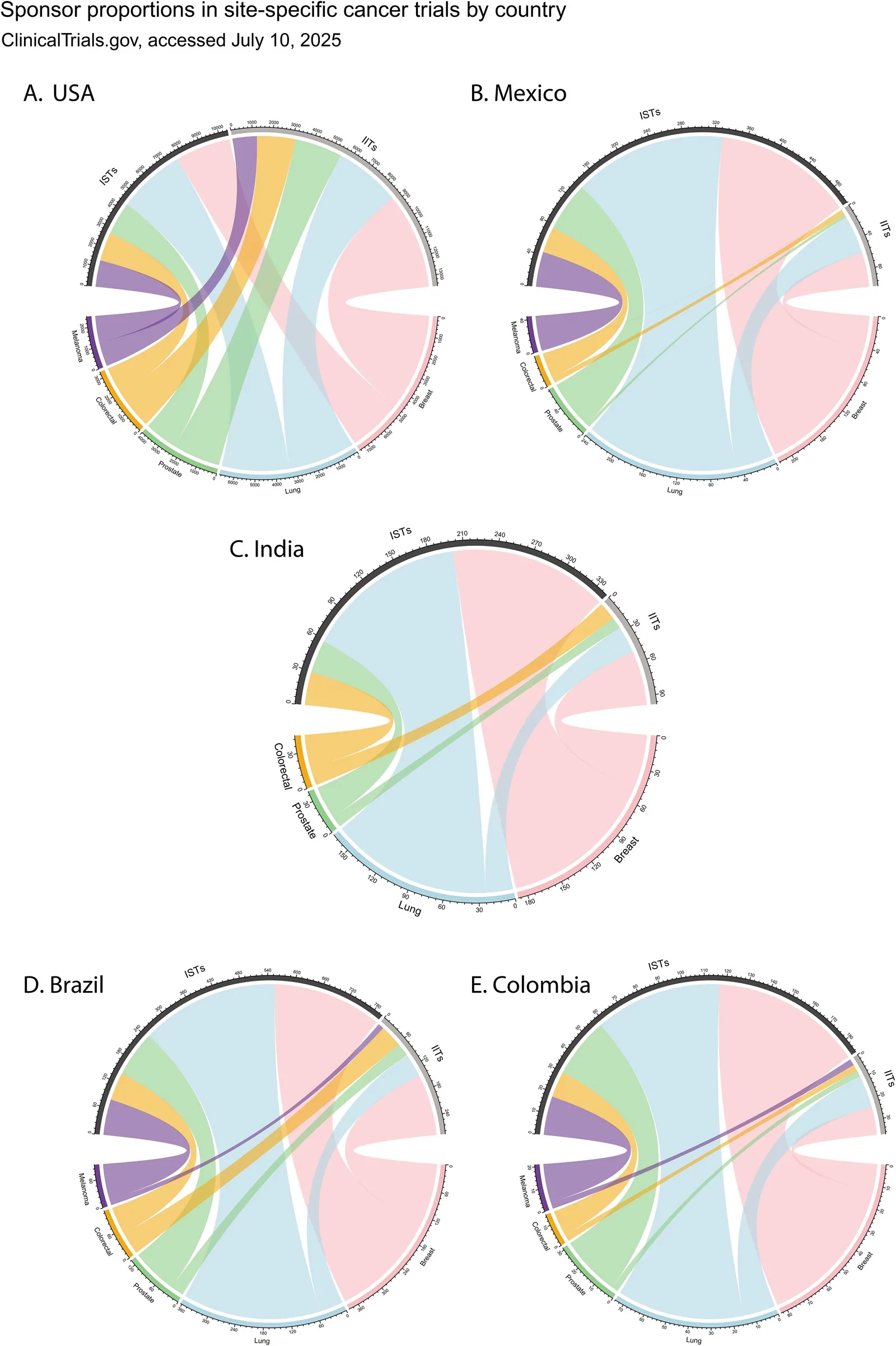
Sponsor composition of registered oncology trials by tumor site, as chord diagrams for (A) United States, (B) Mexico, (C) India, (D) Brazil, and (E) Colombia. Arcs represent the funding categories—Industry (industry-sponsored trials, IST) and Independent (investigator-initiated trials, IIT)—and the pre-specified tumor sites (breast, lung, prostate, colorectal, melanoma; melanoma omitted for India, where only 9 trials, all industry-sponsored, were registered). Ribbon width is proportional to the number of trials, and the analysis is descriptive only. Source: ClinicalTrials.gov, accessed July 10, 2025.

Cancer research distribution by anatomical sites often reflects each country’s disease burden. In the United States, breast and lung cancer trials comprise 29.3% of all cancer studies. The 5 major sites show IIT proportions of 47.9-63.4%, indicating balanced industry-independent research. In LMICs such as India, Mexico, Brazil, and Colombia, industry support dominates at ∼56%-100% across major cancers **(**Table 1 and Figure 3**)**. Thus, LMIC trials cluster on the same high-incidence cancers as high-income countries, yet may leave locally important questions, such as de-escalation, affordability, and implementation, underexplored. This pattern points to a clear opening for public or philanthropic investment in IITs targeting those gaps.

**Table 1.** Distribution of cancer clinical trials at ClinicalTrials.gov by site and funding source in the United States, Brazil, Mexico, India, and Colombia, as of July 10, 2025.

Country	Cancer site	Total trials	ISTs (%)	IITs (%)
**United States**	Breast	7465	2733 (36.6)	4732 (63.4)
	Lung	6670	3357 (50.3)	3313 (49.7)
	Prostate	4000	1628 (40.7)	2372 (59.3)
	Colorectal	3312	1409 (42.5)	1903 (57.5)
	Melanoma	2524	1314 (52.1)	1210 (47.9)
**Brazil**	Breast	382	244 (63.9)	138 (36.1)
	Lung	363	310 (85.4)	53 (14.6)
	Prostate	132	103 (78.0)	29 (22.0)
	Colorectal	113	63 (55.8)	50 (44.2)
	Melanoma	93	80 (86.0)	13 (14.0)
**Mexico**	Breast	217	174 (80.2)	43 (19.8)
	Lung	240	192 (80.0)	48 (20.0)
	Prostate	66	62 (93.9)	4 (6.1)
	Colorectal	39	32 (82.1)	7 (17.9)
	Melanoma	44	44 (100.0)	0 (0.0)
**India**	Breast	188	140 (74.5)	48 (25.5)
	Lung	167	143 (85.6)	24 (14.4)
	Prostate	39	29 (74.4)	10 (25.6)
	Colorectal	45	29 (64.4)	16 (35.6)
	Melanoma	9	9 (100.0)	0 (0.0)
**Colombia**	Breast	83	70 (84.3)	13 (15.7)
	Lung	74	59 (79.7)	15 (20.3)
	Prostate	31	28 (90.3)	3 (9.7)
	Colorectal	15	12 (80.0)	3 (20.0)
	Melanoma	21	18 (85.7)	3 (14.3)

Abbreviations: IITs, investigator-initiated trials; ISTs, industry-sponsored trials.

These imbalances also translate into missed opportunities for scientific innovation. A systematic review revealed that randomized clinical trials from LMICs, although underrepresented in the global literature, were more likely to identify effective interventions and report larger effect sizes compared to those led by HICs.^17^

Even when community-driven questions are addressed by well-conducted, locally or regionally led trials, the resulting studies can struggle to reach high-impact international journals. In our experience, this reflects several factors: structural barriers such as limited funding, thinner mentorship networks, language, and perceptions of limited novelty or generalizability; and, in some submissions, gaps in reporting, statistical rigor, or English-language presentation. The net effect is a visibility gap, in which LMIC research is disproportionately published in local or lower-impact venues despite the methodological expertise and internationally recognized investigators behind much of it.^17^ Encouragingly, several global-health journals now require reflexivity or equitable-partnership statements,^18^ a step toward fairer evaluation, though one that may matter most for low- and lower-middle-income settings. Closing this gap matters not only for equity but because it is necessary to produce decision-grade evidence that is credible to local authorities and transferable across similar health systems.

## From cost to clinical application

Drug development costs remain prohibitive. On average, bringing a single new drug to market is estimated to cost US $2.87 billion.^19^ Oncology clinical research likewise remains concentrated in high-income countries: in a global analysis of phase 3 oncology trials, 92% were led by high-income countries and only 8% by LMICs.^17^

IITs place particular demands on academic groups. In ISTs, the sponsor typically provides staff to handle administrative and regulatory tasks; in academic trials, these responsibilities fall to the research team itself. Although this is true in any setting, the burden is felt acutely in many LMICs, where trained clinician-researchers are scarce and clinical care is prioritized over research.^20^ In Brazil and other LMICs, academic trials must meet the same regulatory requirements as ISTs but without comparable funding or dedicated staff, which can render them financially unsustainable for academic groups.^21^

Translational research bridges the laboratory and the clinic, making preclinical discoveries accessible at the patient’s bedside. However, it depends on sustained laboratory, bioinformatics, and funding infrastructure that remain limited in many LMICs, constraining the local generation and clinical translation of molecular findings.

Collectively, these challenges limit the ability of LMICs to generate timely, high-quality, and contextually relevant evidence. These research gaps restrict access to innovative cancer screening tools, diagnostics, and treatment strategies, which in turn worsen disparities.^22^ For example, the scarcity of region-specific survivorship and supportive-care guidelines leaves a growing population of cancer survivors in LMICs without tailored follow-up, reflecting the broader under-prioritization of cancer as a public-health issue.^23^

## Strengthening independent cancer research in LMICs

Effective cancer control needs input from patients, clinicians, policymakers and civil society. In LMICs, setting regional research and care priorities is crucial for understanding disease patterns in diverse populations. Key determinants, such as epidemiological trends, environmental exposures, genetic profiles, and treatment tolerability, vary significantly between LMICs and HICs. Locally led, independent research is crucial for identifying these differences and informing policies that are contextually relevant and equitable. The results of such research should guide public health authorities and decision-makers in the design and implementation of cancer control strategies.^1^

Translating such priorities into action first requires population-level data. One foundational component of this effort is the establishment of robust cancer registry networks.^24^ Government-funded, independently run population-based cancer registries (PBCRs) supply the epidemiological baseline on which all other cancer-control policies lie. These systems support the evaluation of cancer prevention programs, disease surveillance, and monitoring of survival rates. China’s PBCR network, built over 6 decades and now covering a large share of the population, illustrates this: the incidence and survival data it generates have been used to prioritize national screening programs and inform cancer-control planning.^25^

Data are inert without sustained financial and regulatory support. The development of regional funding mechanisms and philanthropic support must complement sustained public investment. Public–private partnerships, regional cancer research funds, and innovation grants diversify funding and reduce reliance on industry funding. Establishing national cancer research agencies or commissions with protected budgets may also ensure long-term commitment. Moreover, strengthening ethical review processes and streamlining regulatory pathways are essential to fostering a research-conducive environment. Many LMICs face delays due to fragmented or inconsistent regulatory frameworks. Creating central ethics committees and harmonized approval forms speeds reviews and protects patients.

Collaborative international research groups aid independent research in LMICs by providing resources, expertise, and mentorship. In Latin America, groups such as LACOG and CLICaP have advanced understanding of resistance mechanisms and tumor genomics.^12^^,^^26^^,^^27^ In Asia, India’s National Cancer Grid, a network linking more than 300 cancer centers, has transformed care and research by developing resource-stratified treatment guidelines, building multicenter research capacity, and pioneering adaptive health-technology assessment to evaluate cancer interventions and inform public funding decisions.^1^^,^^28^ In sub-Saharan Africa, the African Organization for Research and Training in Cancer and the African Cancer Registry Network have expanded regional research and training capacity and population-based cancer registration, generating the data needed to map the local cancer burden and connect investigators to international collaborations.^29^^,^^30^

A skilled workforce requires training in epidemiology, biostatistics, health economics, clinical trials, and manuscript writing. In many LMICs, researchers in public institutions receive inadequate compensation compared to clinical practice, making research unattractive. Low public-sector salaries drive clinician scientists to private practice; protected time and competitive pay are needed.^21^ Research careers must be both intellectually and financially rewarding. Funding for international conference participation is essential, as LMIC researchers can form alliances, secure funding, and learn emerging methods.

Conventional explanatory trials ask whether a therapy can work under ideal, tightly controlled conditions, whereas LMIC health systems also need to know whether it works in everyday clinical practice. Pragmatic clinical trials (PCTs) address this latter question through broad eligibility, minimal added testing, and outcomes drawn from routine records.^31^ The SWOG S2302 PRAGMATICA-Lung phase 3 trial, led by NCI and academic investigators with pharmaceutical support, used a streamlined pragmatic design to enroll 838 NSCLC patients in 21 months and report results within roughly 2 years of opening.^32^ Such trials demonstrate how academic–industry collaboration can deliver efficient, practice-relevant evidence.

Notably, LMIC investigators were applying pragmatic, real-world approaches well before platforms such as PRAGMATICA-Lung. In Mumbai, a cluster-randomized trial of biennial clinical breast examination in 151,538 women, delivered by trained primary health workers, produced a non-significant 15% reduction in breast-cancer mortality overall and a significant reduction of nearly 30% among women aged ≥50 years, at far lower cost than mammographic screening.^33^ At Tata Memorial Center, an open-label phase III study showed all-oral metronomic chemotherapy was non-inferior to intravenous cisplatin for advanced head-and-neck cancer, with fewer grade ≥3 toxicities and lower drug expenditure.^34^ These independent, publicly oriented trials illustrate how pragmatic IITs can generate inclusive, cost-sensitive evidence directly relevant to LMIC health systems.

Pragmatic trials offer a powerful and realistic pathway for LMICs to answer questions not always explored in oncology trials, such as de-escalation and dose optimization.^31^ By embedding randomization into everyday workflow, shorter durations and reduced dosing can be evaluated efficiently, and less-intensive monitoring can achieve non-inferior survival while meaningfully lowering toxicity, cost, and treatment burden.^35^ Their use of real-world endpoints, such as patient-reported outcomes and healthcare utilization, captures tradeoffs that traditional efficacy trials miss, and adaptive or cluster designs can speed learning across diverse settings. With proportionate regulation, shared data standards, and modest capacity building, they align evidence generation with practice so LMIC systems can determine when “less” is safely “enough.”

This review has limitations. As a narrative review, the initiatives we highlight are illustrative rather than exhaustive, and the focus countries reflect the regional expertise of the author group rather than a systematic selection. Population-based registry research remains limited and unevenly distributed across many LMICs, so registry-derived evidence, and the populations it represents, is underrepresented here. Finally, the bibliometric comparisons draw on PubMed-indexed publications, which under-represent regional-language journals and therefore likely understate Latin American research output.

## Conclusions

The global cancer burden is escalating and will keep rising as the population ages. For LMICs, the challenges of delivering effective cancer care are magnified by structural and socioeconomic constraints. Because industry sponsors most cancer trials in LMICs, research often reflects market priorities more than public-health needs. The perspective we present here draws on the experience of clinicians and scientists embedded in these local health systems.

Local professionals understand how epidemiology, culture, environment, and economics shape patients’ needs. Independent, locally led cancer research is therefore essential to bridge this disconnect. By targeting local challenges, such studies yield actionable evidence that can guide accurate diagnostics, tailored therapies, and more equitable access.

In sum, closing these research gaps starts with empowering LMIC institutions to set and lead their own efforts. That goal needs sustained public funding, cross-disciplinary partnerships, and modern research platforms. Together, these measures will build research infrastructure, train the next generation of investigators, and expand global collaboration, moving the world toward more inclusive and effective cancer control.

## Data Availability

All data analyzed in this review were derived from publicly available sources. The clinical-trial counts were obtained from ClinicalTrials.gov (accessed July 10, 2025), the research-and-development expenditure figures from the UNESCO Institute for Statistics database, and the bibliometric publication counts from PubMed. No new primary patient-level data were generated. The compiled datasets and the search and extraction parameters underlying Table 1 and Figures 1–3 are available from the corresponding author on reasonable request.
